# Design and characterization of a new peptide vector for short interfering RNA delivery

**DOI:** 10.1186/s12951-015-0098-0

**Published:** 2015-06-09

**Authors:** Baoling Chen, Wen Xu, Ran Pan, P. Chen

**Affiliations:** Department of Chemical Engineering, University of Waterloo, 200 University Avenue West, Waterloo, ON N2L 3G1 Canada; Waterloo Institute for Nanotechnology, University of Waterloo, Waterloo, ON N2L 3G1 Canada

**Keywords:** siRNA delivery, RNA interference, Peptide, Cytotoxicity

## Abstract

RNA interference holds tremendous potential as one of the most powerful therapeutic strategies. However, the properties of short interfering RNA (siRNA), such as hydrophilicity, negative charge, and instability in serum have limited its applications; therefore, significant efforts have been undertaken to improve its cellular uptake. Cell penetrating peptides have been utilized to deliver various biologically active molecules, such as proteins, liposomes, nanoparticles, peptide nucleic acids, and recently small interfering RNAs. Here, we introduce a new cell penetrating peptide GL1(Ac-GLWRAWLWKAFLASNWRRLLRLLR-NH_2_) to improve the intracellular uptake of siRNA. This peptide consists of four tryptophan residues that facilitated its binding with the cell membrane, five arginine residues and one lysine residue which are positively charged at physiological pH, which induced the formation of peptide-siRNA complexes and enhanced the affinity of the peptide and cell membrane. Moreover, GL1 adopted helical secondary structure due to the altered distribution of polar and nonpolar residues in the sequence. In this study, we investigated the effect of peptide/siRNA molar ratio on the particle size, surface charge, secondary structure, and uptake efficiency. The results showed that GL1 formed stable complexes with siRNA mainly through electrostatic interaction and hydrophobic interaction, and the complexes displayed a spherical shape with the size of ~100 nm and positive surface charge. Utilizing the techniques of fluorescence microscopy and flow cytometry, the intracellular localization of Cy3-labeled GAPDH siRNA was visualized and the cellular uptake was quantified. It is worth noting that in the serum free environment, compared to Lipofectamine 2000, GL1 achieved higher cellular uptake of siRNA (~95%); in the presence of serum, GL1 retained the same level of siRNA cellular uptake (~84%) as Lipofectamine 2000. In addition, the viability of cells treated by GL1 in all studied molar ratios was >85%, which was significantly higher than that treated by Lipofectamine 2000 (~70%). Taken together, the peptide GL1 demonstrated promise as a siRNA delivery system.

## Background

The discovery of RNA interference (RNAi) has changed the field of gene therapy. RNAi is a post-transcriptional gene silencing process that can specifically and potently knock down the expression of target genes both in vitro and in vivo [1, 2]. RNAi can be induced by short interfering RNA (siRNA). siRNAs are double stranded RNA fragments with 21–23 nucleotides that are capable of inducing the cleavage of mRNAs with the complementary sequence [1–3]. It is currently accepted that RNAi is one of the most important strategies for sequence-specific gene silencing in basic research and a potentially powerful therapeutic treatment for various human diseases [4, 5]. However, siRNA induced RNAi at cellular level is limited by its poor cellular uptake associated with low permeability of the cell membrane to RNA [6]. Thus, an effective and safe transport system to condense and deliver synthesized siRNA into cells is mandatory. During the last two decades, a new family of peptides known as cell-penetrating peptides (CPPs) were identified and thoroughly studied. CPPs present the ability to cross cellular membranes by a remarkably low toxic process and to promote the cellular internalization of biomolecules such as peptides, proteins, nucleic acids, peptide nucleic acids (PNA), liposomes, and nanoparticles [7–10]. However, the efficacy of siRNA delivery mediated by CPPs remains unsatisfactory, mainly due to the insufficient delivery efficiency, cytotoxicity, and/or sensitivity to the medium [6].

In order to increase the potency of such vectors, we designed a new peptide GL1. The following criteria were considered for the peptide design and its sequence contains: (1) hydrophobic residues, such as tryptophan, to facilitate membrane binding owing to its strong preference for the lipid membrane interfacial region [11]; (2) cationic residues, such as arginine and lysine, to assist the formation of peptide-siRNA complexes and peptide-membrane interactions through electrostatic interaction and hydrogen bonding [11]; (3) altered distribution of the polar and nonpolar residues, to achieve better interaction with the cell membrane due to the formation of helical secondary structure [12]. The prevailing hypothesis is that the cationic peptide GL1 electrostatically complexes with the anionic siRNA to produce a neutral or positively charged complex that has sufficient stability to allow intracellular delivery of siRNA. In this study, we investigated the physicochemical characteristics of GL1 when interacting with siRNA and evaluated the effect of its physicochemical characteristics on siRNA delivery efficiency and cytotoxicity. Chinese hamster ovary (CHO-K1) cells are a cell line derived from the ovary of the Chinese hamster and often used in studies of genetics, toxicity screening, and nutrition and gene expression because of its rapid growth rate. Thus, CHO-K1 was used as a model system to test the biological responses for siRNA delivery in this study.

## Methods

### Materials

The GL1 peptide (Ac-GLWRAWLWKAFLASNWRRLLRLLR-NH_2_) with a molecular weight of 3,123.8 g/mol, was synthesized by CanPeptide Inc. (Quebec, CA) with 95% purity. Cy3-labeled GAPDH siRNA was purchased from Ambion (SilencerTM GAPDH siRNA kit). The siRNA targeting sequence for eGFP (GCGACGUAAACGGCCACAAGU) was purchased from Dharmacon. The sense sequence is GACGUAAACGGCCACAAGUUC and the antisense sequence is ACUUGUGGCCGUUUACGUCGC. The negative control siRNA used here was purchased from Ambion. The MTT (3-(4,5-dimethylthiazol-2-yl)-2,5-diphenyltetrazolium bromide, a yellow tetrazole) assay kit was purchased from Sigma-Aldrich (Oakville, CA).

### Cell line

Chinese hamster ovary (CHO-K1) cells were purchased from American Type Culture Collection (ATCC CCL-61). The cells were cultured in F-12K medium (Thermo Scientific, Ottawa, Canada) with 10% fetal bovine serum (FBS) (Sigma-Aldrich, Oakville, Canada). The cells were incubated at 37°C in a humidified atmosphere with 5% CO_2_.

### Preparation of GL1 and GL1-siRNA complexes

The peptide in powder was dissolved in filtered RNase-free water at a concentration of 500 µM, vortexed for 5 s, and ultrasonically mixed for 5 min. This stock was diluted to the desired concentrations for experiments. GL1-siRNA complexes were formed by simply mixing GL1 solutions to siRNA solutions at desired concentrations and ratios, and incubated at room temperature for 20 min.

### Gel electrophoresis

The GL1-siRNA complexes in RNase-free water were made with 300 ng of eGFP siRNA. The samples of siRNA alone and GL1-siRNA complexes at molar ratios (peptide/siRNA) ranging from 1/1 to 80/1 were electrophoresed on a 1.2% wt/vol agarose gel in 1× TBE at 55 V for 1 h. The ethidium bromide-stained siRNA was visualized on an ultraviolet transilluminator with a camera.

### Isothermal titration calorimetry (ITC)

The isothermal titration calorimetry experiments were conducted using a Nano-ITC calorimeter (TA Instruments). A 500 μM GL1 peptide solution and a 10 μM siRNA solution were both prepared in RNase-free water. All of the samples were degassed in a degassing station (TA Instruments) prior to the experiments. RNase-free water was placed in the ITC reference cell. For each titration, 2 μl of the peptide in a pipette rotating at 250 rpm was injected into the siRNA solution in the sample cell of the calorimeter, which was equilibrated to 25°C, with an interval of 300 s between injections. The heat of dilution was measured by titrating the GL1 solution into RNase-free water and was later subtracted from the sample measurement. The data were analyzed using NanoAnalyze software v.3.1.2.

### Size and zeta potential measurements

Particle size measurements were done with a Zetasizer Nano ZS (Malvern, United Kingdom) with transparent ZEN0040 disposable microcuvette cells (40 µl) at 25°C. The GL1-siRNA samples were prepared as mentioned above with a siRNA concentration of 100 nM. The zeta potentials were measured in a Clear DTS1070 zeta dip cell with the same machine. For zeta potential measurement in serum conditions, GL1-siRNA complexes were freshly prepared and diluted 1:10 with F12K + 10% FBS. The complexes were incubated in a serum-supplemented medium for 10 min before the measurement.

### Scanning electron microscopy (SEM)

The GL1-siRNA complexes were imaged with a SEM to characterize their morphology. The samples were prepared as mentioned in “Cellular uptake and localization of siRNA complexes” with a siRNA concentration of 100 nM and a GL1 to siRNA molar ratio 40/1. After the sample was incubated for 20 min at room temperature, 40 µl of the sample placed on conductive silicon wafer was allowed to dry at room temperature. The sample was coated with 10 nm-thick gold before imaging. The image was taken using a LEO FESEM 1530 field-emission SEM.

### Circular dichroism (CD) spectroscopy

Spectra at 250–190 nm with a spectral resolution and pitch of 1 nm and scan speed of 200 nm/min were recorded using a J-810 spectropolarimeter (Jasco, USA). Increasing amounts of siRNA were added to the peptide, which was at a fixed concentration of 30 μM, to obtain different molar ratios of the two compounds. After 20 min incubation, the samples were transferred into 1-mm long quartz cells and maintained at 25°C. The spectra presented here are the average of three measurements.

### Fluorescence microscopy and fluorescence-activated cell sorting (FACS)

CHO-K1 cells were seeded at a density of 50,000 cells per well and incubated for 24 h in a 24-well flat-bottom plate before treatment. The cells were incubated with GL1-Cy3-labeled siRNA complexes at a molar ratio of 20/1 in OPTI-MEM (Invitrogen) for 4 h. Thereafter, the cells were rinsed with phosphate buffered saline (PBS) and washed with heparin (10 U/ml). After, these cells were fixed with 500 μl of cold 4% PFA for 30 min and then washed twice with PBS. The nuclei were stained using a DAPI solution (Sigma-Aldrich, Oakville, CA, USA). Images were taken with an inverted fluorescence microscope (Zeiss AxioObserver Z1, CA, USA) and analyzed using AxioVision software.

The uptake of Cy3-labeled siRNA into the CHO-K1 cells was quantified with a BD FACSVantage SE Cell Sorter flow cytometry (BD Biosciences, United States). CHO-K1 cells were plated at a density of 50,000 cells per well and incubated for 24 h in a 24-well flat-bottom plate. The medium was replaced with GL1-siRNA complexes and control samples in OPTI-MEM (Invitrogen) or F12-K supplemented with 10% FBS with a siRNA concentration of 100 nM. After 4 h of incubation, the cells were rinsed with phosphate buffered saline (PBS) and washed with heparin (10 U/ml). After, trypsin–EDTA was added to detach the cells from the plate. The cells were re-suspended in 4% paraformaldehyde (PFA) and ready for quantitative analysis.

### In vitro gene silencing efficiency

CHO-K1 cells were seeded at 35,000 cells/well in 24-well plates and incubated for 24 h. Next, the cells were incubated with various complex formulations in Opti-MEM at 37°C for 4 h. Then, the cells were incubated for an additional 48 h in complete culture medium with siRNA concentration of 50 nM. To determine the serum effect on gene silencing efficiency, the cells were directly incubated with various complex formulations in complete culture medium for 52 h and then prepared for analysis.

For qRT-PCR analysis, the total RNA was extracted from the treated cells using the SV Total RNA Isolation System (Promega, CA, USA). A Nanodrop (Nanodrop spectrophotometer ND-1000, Thermo Scientific, Ottawa, CA) was used to determine the RNA concentrations. The RNA samples were reverse-transcribed into cDNA using a Bio-Rad iScript cDNA synthesis kit according to the manufacturer’s protocol. After the cDNA was synthesized, PCR was performed with Brilliant II fast SYBR Green QPCR Master Mix (Agilent Technologies, Wilmington, DE, USA) using an Mx3005P™ real time PCR System (Agilent Technologies). The sequences of the primers used for the mouse GAPDH gene are 5′-TTGCTGTTGAAGTCGCAGGAG-3′ and 5′-TGTGTCCGTCGTGGATCTGA-3′ (Sigma, Oakville, Ontario, Canada). Cyclophilin, a house-keeping gene, was used as an internal control to normalize the GAPDH gene expression. Mouse cyclophilin mRNA amplified using the following primers: 5′-AGGGTTTCTCCACTTCGATCTTGC-3′ and 5′-AGATGGCACAGGAGGAAAGAGCAT-3′ (Sigma, Oakville, Ontario, CA, USA).

### Cytotoxicity assay

The cytotoxicity of peptide-siRNA complexes was determined by the MTT assay. In brief, cells were seeded at 5,000 cells/well in clear, flat-bottomed, 96-well plates (Costar) 24 h before treatment. After being washed, 100 μl of Opti-MEM that contained peptide-siRNA complexes at different molar ratios was added to the wells and incubated for 4 h. Thereafter, 50 μl of 30% serum containing medium was added, and the cytotoxicity of the relevant reagents was determined by the MTT assay after 48 h. The absorbance at 570 nm was read on a plate reader (FLUOstar OPTIMA, BMG, Germany). The background absorbance of the multiwell plates at 690 nm was determined and subtracted from the 570 nm measurement. The results obtained from triplicate wells were averaged and normalized to the value obtained from the non-treated cells.

### Statistical analysis

Results were expressed as mean values ± SD. Data were analyzed by two tailed T test and only p values <0.05 were considered statistically significant.

## Results and discussion

### Effect of peptide to siRNA molar ratio on complex formation

**Figure 1:**
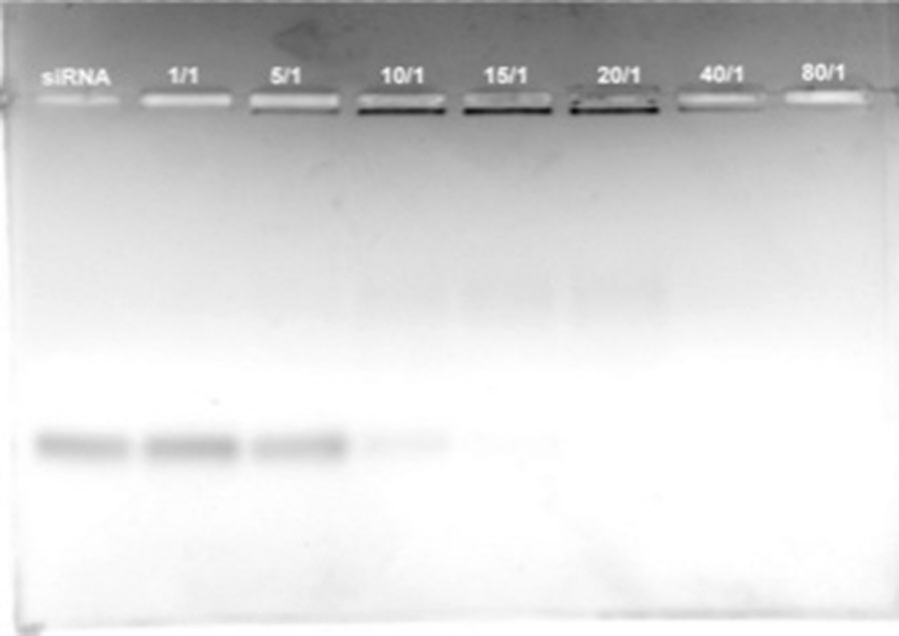
Binding ability of siRNA to GL1 studied by agarose gel-shift assay The formed GL1-siRNA complexes, stained with ethidium bromide, were investigated by electrophoresis on agarose gel (1.2% wt/vol). siRNAs, targeting eGFP genes, were complexed with GL1 at a series of molar ratios from 1/1 to 80/1. *Lane* 1 was siRNA control, and *lanes* 2–8 indicated correlated molar ratios. The amount of siRNA was 300 ng.

**Figure 2:**
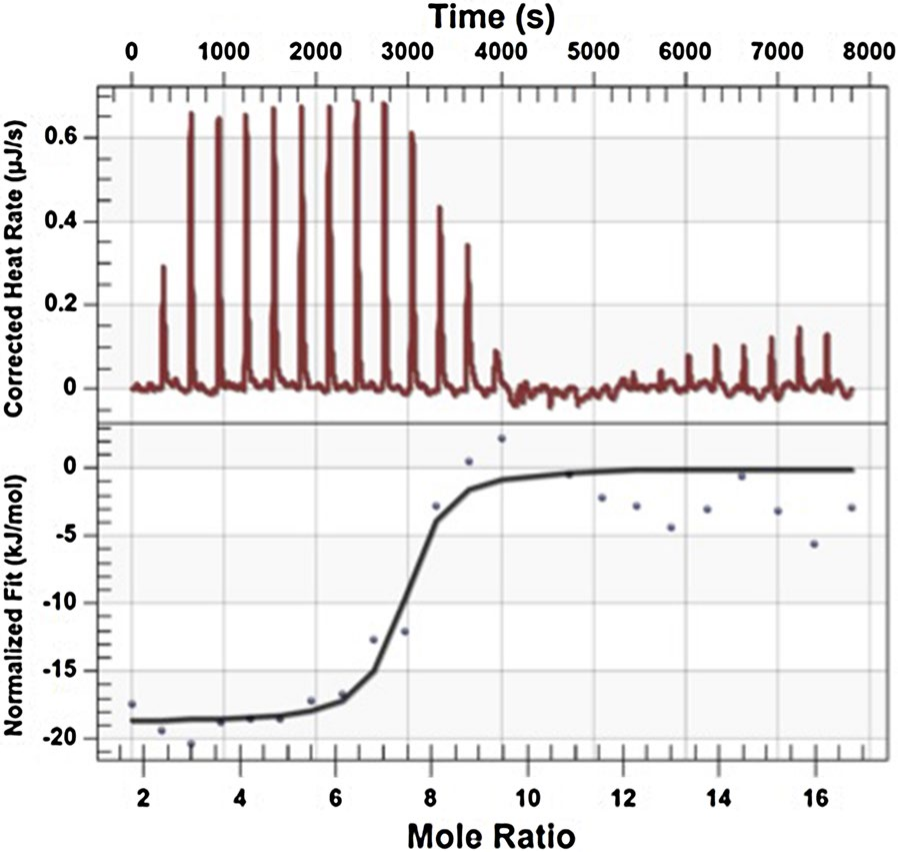
Calorimetric titration of siRNA with GL1 at 25°C in RNasefree water at pH 6. **a** Corrected thermogram of calorimetric titration of siRNA with GL1. **b** Binding analysis of siRNA with GL1 by fitting the raw data with an independent model. GL1 concentration was 500 μM and siRNA concentration was 10 μM.

The ability of GL1 to interact with siRNA was investigated by gel electrophoresis. Binding between peptide and siRNA prevented RNA from being stained by EtBr. As shown in Figure 1, as molar ratio increased, the intensity of siRNA decreased sharply due to the formation of GL1-siRNA complexes. At molar ratio 10/1, the intensity of siRNA was significantly low. At a slightly higher molar ratio 15/1, no free siNRA was detected on the agarose gel. This result indicated that the minimal molar ratio for siRNA delivery was around 10/1. This direct binding between cationic peptide and anionic siRNA would be initiated via electrostatic interactions. The formation of GL1-siRNA complexes and the thermodynamic parameters associated with the binding between peptide and siRNA was further investigated using isothermal titration calorimetry by titrating the siRNA with GL1. The heat exchange during the titration of siRNA by GL1 (in RNase-free water) was detected by the machine and output as raw data. By fitting the raw data (upper panel of Figure 2) to a single-site model (lower panel of Figure 2), the thermodynamic parameters during the interaction were obtained and listed in Table 1.

**Table 1:** Thermodynamic parameters when titrating siRNA with GL1 in water

Ka (1/M)	ΔH (kJ/mol)	n	K_d_ (M)	ΔS (J/(mol K))
4.47 × 10^8^	−18.71	7.16	2.24 × 10^−7^	64.56

GL1 represents high affinity for siRNA with the dissociation constant of 2.24E−7 M. The obtained molar stoichiometry was 7.16, which implied that 7.16 mol GL1 could condense 1 mol siRNA. Theoretically, 7 mol GL1 could condense 1 mol siRNA, since the GL1 peptide contains six positively charged residues (Arginine and Lysine) and the siRNA molecule consists of 21 pairs of negatively charged nucleotides. The experimental data was consistent with the theoretical value. In the ITC experiment, due to the stirring throughout the titration process, peptide and siRNA could interact to its full extent. However, in the gel experiment, the peptide and siRNA solution was mixed first and then incubated for 20 min without further mixing. The difference in the experimental settings leads to the slight difference in the molar stoichiometry. With an enthalpy of −18.71 kJ/mol, ΔS of 64.56 J/(mol K), and entropy of −19.24 kJ/mol, the binding was entropy-driven, but enthalpy was also a major factor. Moreover, through calculation using the equation ΔG = ΔH − TΔS, ΔG was −37.94 kJ/mol indicating that the formation of GL1-siRNA complexes was a thermodynamically favored process [13, 14].

### Particle size and zeta potential

**Figure 3:**
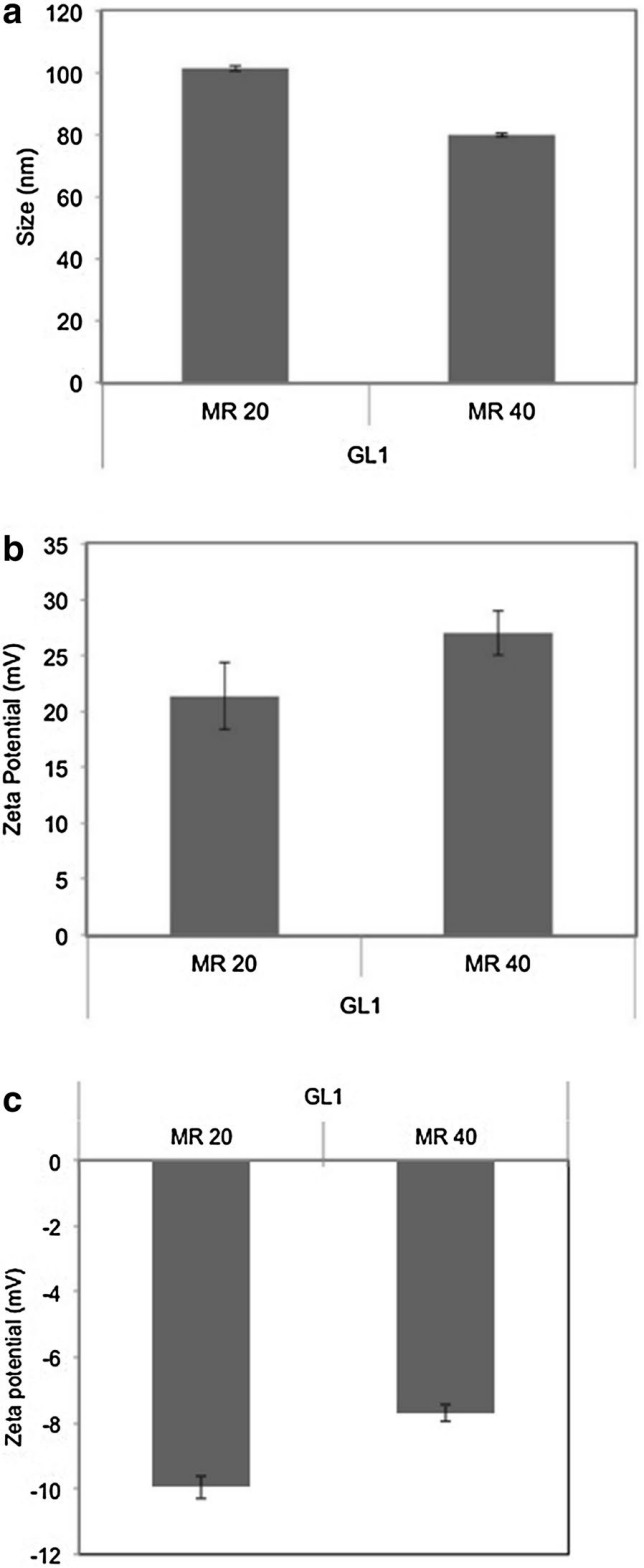
**a** The hydrodynamic diameter and **b** zeta potential of the GL1-siRNA complexes prepared in RNase free water at different molar ratios were measured by dynamic light scattering (DLS); **c** zeta potential of the GL1-siRNA complexes in serum-supplemented F12K media. The siRNA concentration was fixed at 100 nM. At different molar ratios, the amount of GL1 was adjusted. Results are expressed as mean ± standard deviation (n = 3).

Considering that particle size and zeta potential are important characteristics of delivery systems associated with the delivery efficiency [15], dynamic light scattering was applied to gain a view of the physicochemical properties of GL1-siRNA complexes. It has been reported that 200 nm is the approximate limit for cellular uptake by macropinocytosis and nanoparticles larger than 200 nm may hinder its cellular internalization [16, 17]. As shown in Figure 3a, particle sizes remained in the range of 80–100 nm, which fell in the range suitable for cellular uptake. Interestingly, at molar ratio 40/1, particle size slightly decreased. Previous studies prove that as molar/charge ratio increased, stronger electrostatic repulsion between complexes occurs, leading to smaller nanoparticles [18, 19]. The other possible reason is that excess peptides formed smaller nanoparticles, thus decreased the average size. It is generally accepted that nanoparticles with positive surface charge induce uptake via electrostatic interaction with the anionic cell membranes [20]. Figure 3b shows that at molar ratio 20/1 and 40/1, nanoparticles displayed positive surface charge and the absolute values increased at higher molar ratio due to the addition of more cationic peptides. This positive surface charge allows interaction of GL1-siRNA complexes with the polyanionic glycosaminoglycans on the cell surface [21]. However, in serum-containing media, GL1-siRNA complexes exhibited a negative surface charge, indicating that serum proteins interacted with the outer layer of the complex and induced a negative surface charge. This result suggests that in serum conditions the uptake of GL1-siRNA complexes may be mediated by scavenger receptors, which were involved in the cellular uptake of negatively charged macromolecules [22].

### Morphology and secondary structure

**Figure 4:**
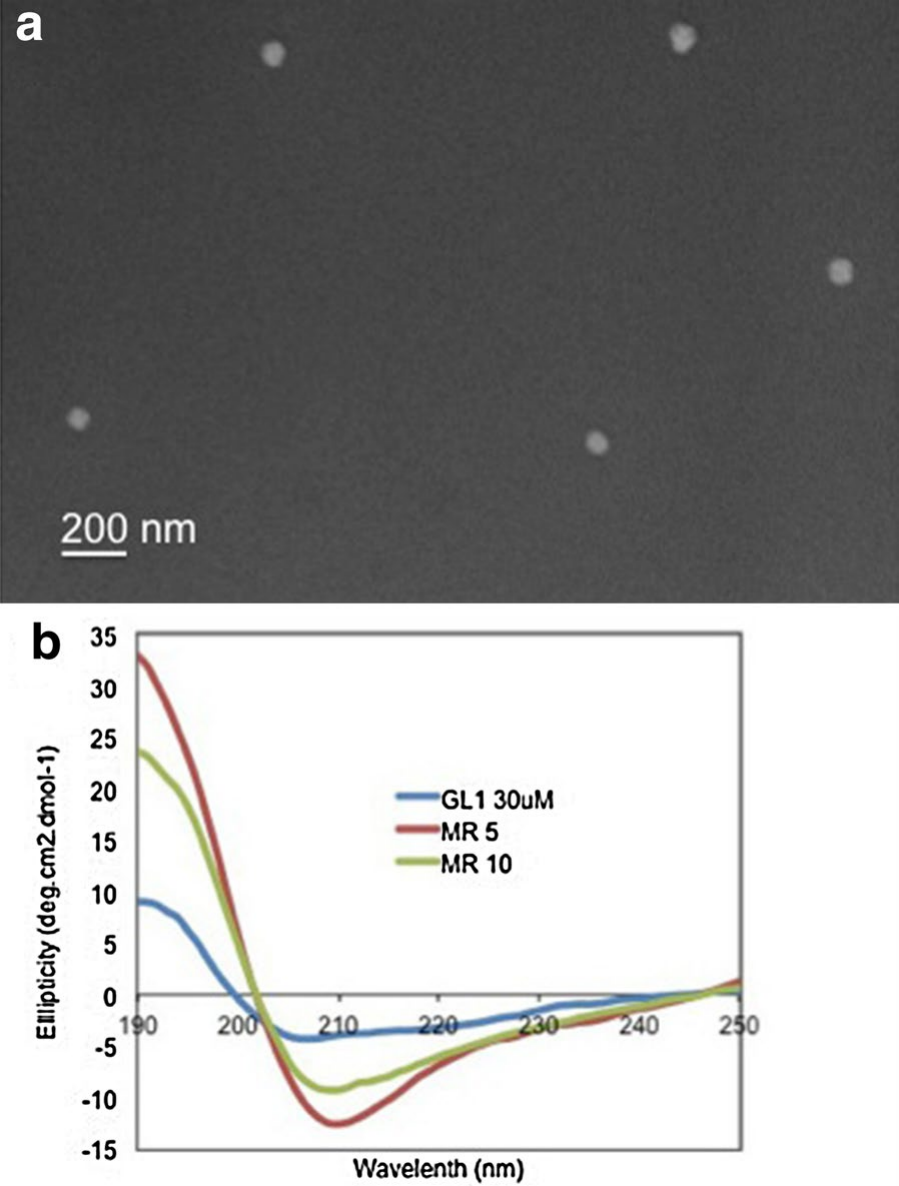
**a** SEM image of GL1-siRNA complexes at molar ratio 40/1, siRNA concentration was 100 nM. **b** CD spectra of GL1 alone and GL1-siRNA at different molar ratios. GL1 concentration was fixed at 30 μM and GL1-siRNA complexes were formulated at molar ratios of 5/1 and 10/1.

Moreover, Figure 4a shows the morphology of the GL1-siRNA complexes formed at molar ratio 40/1 observed under SEM. The SEM image shows that GL1 and siRNA form spherical nanoparticles with the size less than 100 nm. As shown in Figure 4b, The CD-spectrum of GL1 is characterized by a maximum at 190 nm, a minimum at 207 nm, and a slight shoulder at 220 nm which implies that GL1 adopts α-helical structure. Upon adding siRNA to retain the peptide/siNRA molar ratio 10/1, GL1 CD-spectrum displayed a minimum at 210 nm, a maximum at 190 nm, a slight shoulder at 220 nm, and the absolute values of the minimum and maximum increased, which reveals that GL1 adopts a higher content of α-helical structure. Adding more siRNA to retain the peptide/siRNA molar ratio at 5/1, GL1 represented a similar CD-spectrum and the absolute values of the minimum and maximum further increased. Similar phenomena have been reported for other CPPs [23] and this conformational change is due to the presence of anionic component, siRNA, which can screen the positive charge of arginine residues in one face of the helical structure, thus, forming a stable helical structure. This stable helical structure facilitates better interaction with cell membranes and enhances cellular internalization [24].

### Cellular uptake and localization of siRNA complexes

**Figure 5:**
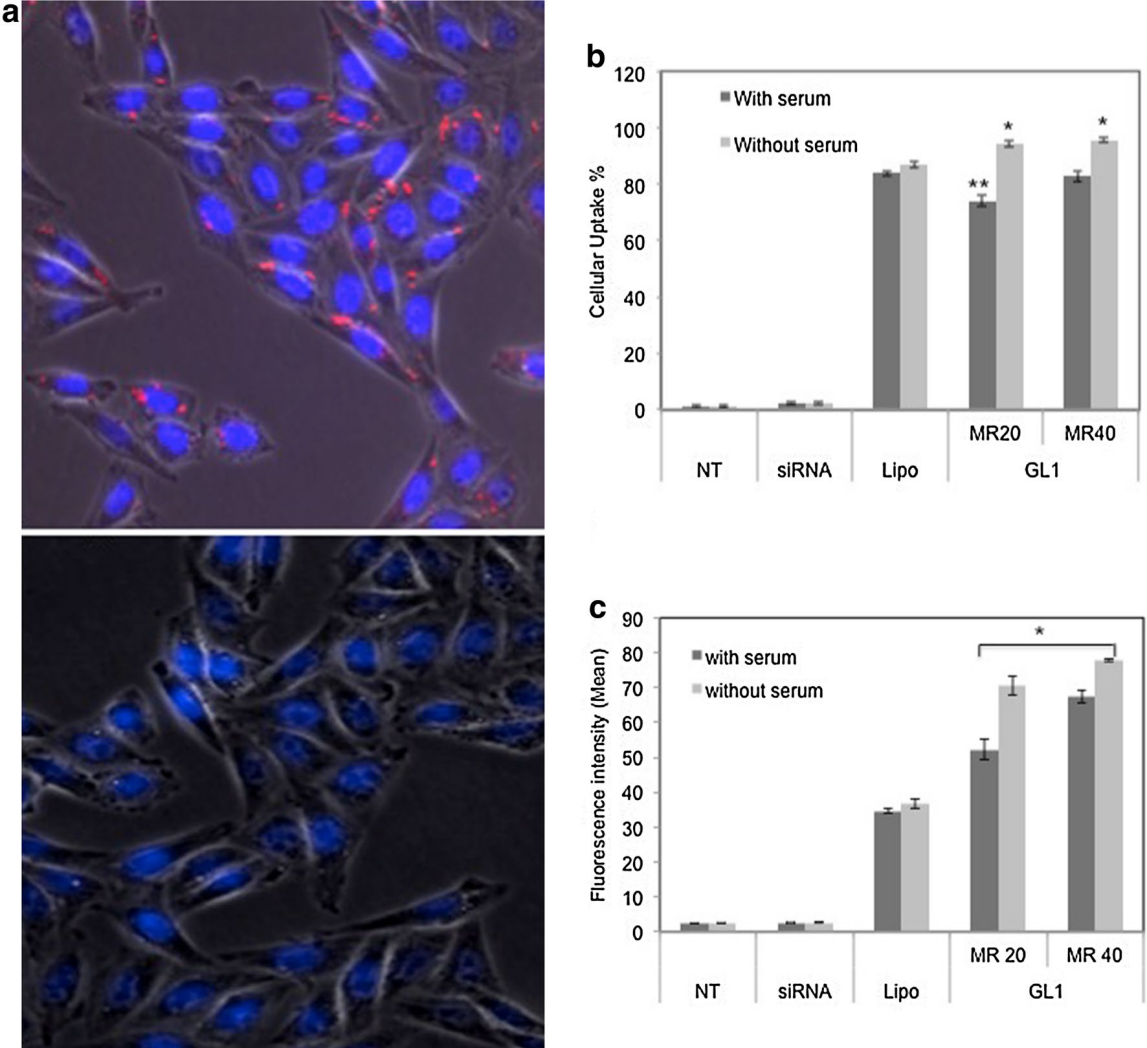
**a** Fluorescence microscope image of GL1-siRNA complexes at molar ratio 40/1 (magnification 40×). The *red fluorescence* indicated Cy3 labeled siRNA, *blue fluorescence* represented DAPI stained nuclei. The *lower panel* was non-treated cells, used as a control. **b** FACS results of cellular uptake of siRNA. *P value < 0.05, in a serum free environment, the uptake of siRNA at molar ratios of 20/1 and 40/1 is significantly different from that after Lipo treatment. **P value < 0.05, in a serum environment, the uptake of siRNA at a molar ratio of 20/1 is significantly different from that at a molar ratio of 40/1 and after Lipo treatment. **c** Relative fluorescent intensities at different treatment conditions. *P value < 0.05, the fluorescence intensity at molar ratios of 20/1 and 40/1 with or without serum is significantly different from that after the other treatments. Non-treated sample was negative control; Lipo-siRNA complexes were positive control. Cy3 labeled GAPDH siRNA was used here. siRNA concentration was 100 nM in both experiments. *NT* non-treated, *MR* peptide/siRNA molar ratio, *Lipo* Lipofectamine 2000. Results are expressed as mean ± standard deviation (n = 3).

The cellular localization of siRNA was shown in Figure 5a. The siRNAs were localized in cytosol and to regions in close proximity to the nuclear membrane and were distributed in a non-homogeneous pattern at the periphery of the nucleus. Previous studies demonstrated that localization of siRNA [25], Dicer [26], and RNAi activity [27] was restricted to the cytoplasm and siRNA entry into living cells was localized to perinuclear regions [28]. Our data indicating that siRNA localization was perinuclear (Figure 5) was in consonance with the previous studies.

siRNA cellular uptake was quantified by FACS. As shown in Figure 5b, c, either with or without the presence of serum, siRNA alone represented almost no uptake in CHO-K1 cells, which also confirmed the limitation of siRNA alone in cellular uptake. Without the presence of serum, peptide GL1 achieved 95% uptake of siRNA at molar ratio 20/1 and 40/1, which is higher than Lipofectamine 2000 (Lipo 87%), one of the most efficient and commercially available transfection reagents. Due to the serum effect, the uptake of siRNA decreased to 75% (molar ratio 20/1) and 84% (molar ratio 40/1). However, at molar ratio 40/1, GL1 still achieved the same level of uptake as Lipo with the presence of serum. However, with or without serum, GL1-siRNA treated cells exhibited higher fluorescence intensity than Lipo and fluorescence intensity increased with the peptide/siRNA molar ratio. This result indicated that peptide GL1 could effectively protect siRNA and efficiently deliver siRNA to cells even in the presence of serum.

### Gene silencing efficiency in vitro

**Figure 6:**
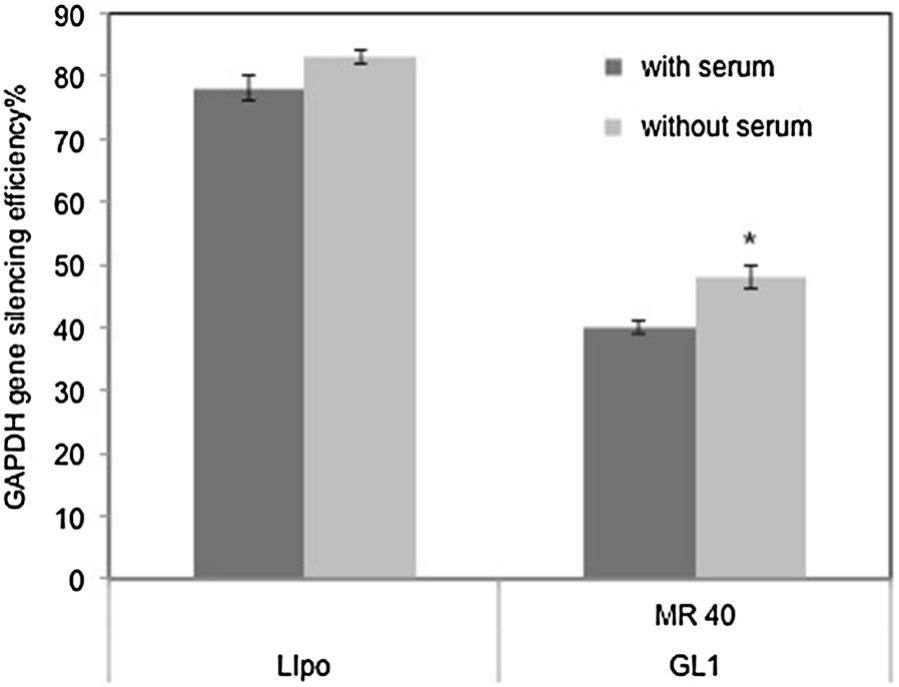
Gene silencing efficiency in vitro. Silencing of GAPDH gene in CHO-K1 cells was evaluated by quantitative real time polymerase chain reaction (qRT-PCR). GAPDH siRNA concentration was 50 nM. Lipo was the positive control, and scrambled siRNA was used as the negative control. Results are expressed as mean ± standard deviation (n = 3). *P value <0.05, the gene silencing efficiency at a molar ratio of 40/1 in a serum free environment is significantly different from the other treatments.

With or without serum, the GAPDH gene silencing efficiency induced by GL1-siRNA complexes (MR 40/1) at the mRNA level was investigated using qRT-PCR. Lipofectamine 2000 was used as a positive control. As shown in Figure 6, GL1-siRNA complexes at a molar ratio of 40/1 achieved 58 and 47% gene silencing efficiency with and without serum respectively. This result indicated that GL1 could efficiently deliver siRNA to cells and induced the specific gene silencing. With serum, gene silencing efficiency of GL1-siRNA complexes decreased compared to that without serum, respectively. This trend was consistent with the cellular uptake result. The Lipofectamine 2000-siRNA complexes induced 80 and 74% GAPDH gene silencing with and without serum. The possible reason that GL1-siRNA complexes did not achieve similar gene silencing efficiency was that the complexes were retained inside endosomes following endocytosis [29].

### Cellular toxicity

**Figure 7:**
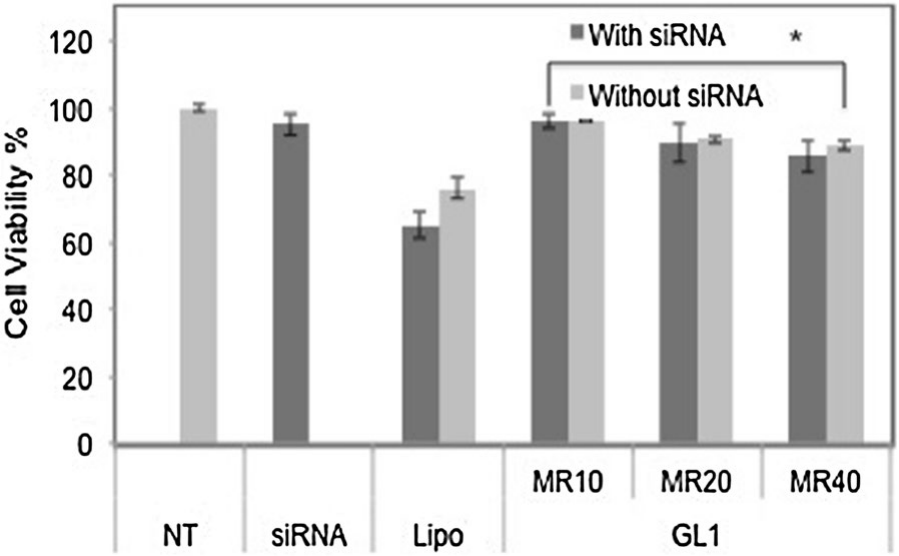
Cell viability results of CHO-K1 cells treated with naked Lipofectamine 2000 or GL1, and the complexes with siRNA (50 nM) at different molar ratios. *NT* non-treated, *MR* peptide/siRNA molar ratio, *Lipo* Lipofectamine 2000. Results are expressed as mean ± standard deviation (n = 3). *P value < 0.05, the cell viability after GL1 treatment is statistically significant than that after Lipo treatment.

For a CPP to be a functional vector, it must achieve high uptake efficiency and exhibit low levels of cytotoxicity. It is well documented that the molar ratio of the positive vector to negative nucleic acid cargo affects toxicity and higher molar ratios may cause higher toxicity [30]. As shown in Figure 7, siRNA alone was not toxic. At molar ratio 10/1, GL1-siRNA complexes and GL1 alone did not cause toxicity. At molar ratio ≥20/1, viability of cells treated either with GL1-siRNA complexes or GL1 alone had a slight drop owing to relatively higher surface charge, but it was still above 85%. Moreover, Lipo alone and Lipo-siRNA complexes achieved lower cell viability ~70%. Thus, peptide GL1 showed attractive characteristics with regards to the criteria of cytotoxicity.

Here, an amphipathic, cationic peptide GL1 was designed and investigated. Structurally, GL1 has dispersed cationic amino acids along the sequence, which differs from the arginine rich peptide R9, R9-hLF, and Tat [31]. The peptide MPG and MPG-like peptides consist of a hydrophobic domain and a hydrophilic domain which contributes to their amphipathic property [32, 33]; while PF6 and PF14 incorporated a stearyl moiety in their sequences to improve amphiphilicity. However, peptide GL1 has hydrophilic and hydrophobic amino acids dispersed along the sequence, which leads to its amphipathicity and helical secondary structure. Another significant difference of GL1 from the peptides mentioned above is that GL1 utilizes four tryptophan amino acids distributed along the sequence to enhance cellular uptake of GL1-siRNA complexes via strong peptide-membrane interaction. Gel electrophoresis results show that GL1 can form complexes with siRNA and encapsulate all the free siRNA at molar ratio 15/1. However, hLF, R9, R9-hLF, Tat, TP10, PF6, and PF14 require much higher molar ratio, near 50/1 to encapsulate all the free siRNA [31]. The ITC data further proved that GL1 had a strong affinity with siRNA (K_d_ = 2.24E−7 M) and the interaction of GL1 with siRNA was thermodynamically favored (ΔG = −37.94 kJ/mol). Additionally, a similar value of Gibbs free energy was reported in our previous study of an amino acid pairing peptide C6 [34]. CADY, a 20-residue secondary amphipathic peptide also encapsulated all the siRNA at molar ratio 15/1 and adopted helical secondary structure. Compared to GL1, CADY displayed even stronger binding affinity with siRNA (K_d_: 0.152E−7 M) [35]. Interestingly, CADY enters cells not through the endosomal pathway but GL1 does, which may lead to the difference in the efficiency of intracellular release of siRNA.

The size of GL1-siRNA complexes slightly decreased from 100 to 80 nm when increasing the molar ratio from 20/1 to 40/1 possibly due to stronger electrostatic repulsion at higher molar ratio. Moreover, GL1-siRNA complexes displayed positive surface charge at a molar ratio of 20/1 (~21 mV). At a molar ratio of 40/1, due to the addition of more cationic peptide, GL1-siRNA complexes’ surface charge increased to 27 mV. Interestingly, GL1 adopted a higher content of helical structure upon interaction with siRNA, possibly due to the decrease of the charge repulsion between cationic amino acids after partial positive charge was neutralized by anionic siRNA. The positive surface charge and secondary structure change suggest that GL1 and siRNA form stable complexes mainly through the intermolecular binding and electrostatic interaction, which was consistent with some reported CPPs [36]. These favourable physicochemical characteristics lead to the significant cellular uptake of Cy3-labeled siRNA (95%), which is higher than Lipo (87%) in a serum free environment. In the presence of serum, GL1-siRNA complexes exhibited negative charge. This is possibly because excess anionic serum protein neutralizes the positive surface charge of GL1-siRNA complexes and displays negative charge. Due to the serum effect, cellular uptake of GL1-siRNA complexes dropped to 85%, which is still comparable to Lipo. Moreover, GL1-siRNA complexes induced 49 or 40% GAPDH gene silencing with serum or without serum respectively, with minimal cytotoxicity. Taken together, our experimental data strongly supported our hypothesis that the cationic peptide GL1 electrostatically complexes with the anionic siRNA to produce a positively charged complex that has sufficient stability to allow efficient intracellular delivery of siRNA and induce specific gene silencing efficiency. Note that GL1 delivered higher amount of siRNA to cells than Lipo but induced lower gene silencing efficiency than Lipo, possibly due to insufficient endosomal release of siRNA. Further modifications of GL1 to enhance endosomal release are required.

## Conclusions

In this study, we have investigated the physicochemical characteristics of the peptide GL1, and evaluated its siRNA delivery efficiency and cellular toxicity on the CHO-K1 cell line. The major conclusions include the following: GL1 could form complexes with siRNA cargo at molar ratio ~10/1 mainly via electrostatic interaction and hydrophobic interaction; GL1 could spontaneously condense siRNA to form spherical particles with sizes on the nanoscale (80–100 nm); GL1 adopted α-helical secondary structure and became more so upon interaction with siRNA molecules to form stable complexes; at molar ratio 40/1, GL1 achieved higher cellular uptake of Cy3-labeled siRNA (95%) than Lipofectamine 2000 in serum free environment and retained the same level of uptake as Lipofectamine 2000 (84%) with the presence of serum; in all the studied molar ratios, GL1 achieved higher cellular viability (>85%) compared to Lipofectamine 2000 (~70%). Therefore, GL1 demonstrated the potential for future applications as a vector for siRNA delivery.

## Abbreviations

siRNA: short interfering RNA
RNAi: RNA interference
CPPs: cell-penetrating peptides
PNA: peptide nucleic acids
CHO-K1: Chinese hamster ovary
FBS: fetal bovine serum
ITC: isothermal titration calorimetry
SEM: scanning electron microscopy
CD: circular dichroism
FACS: fluorescence-activated cell sorting
PFA: paraformaldehyde
PBS: phosphate buffered saline
Lipo: Lipofectamine 2000
